# Supplementary material for: Diagnostic value of long noncoding RNAs as biomarkers for Ankylosing Spondylitis: A systematic review and meta-analysis
Source: PLoS One. 2025 Jul 28;20(7):e0328249. doi: 10.1371/journal.pone.0328249 (PMC12303277; doi:10.1371/journal.pone.0328249)
Supplement: S1 Table — (DOCX) [file pone.0328249.s002.docx]

**S1 Table:** **Search strategy used to retrieve eligible studies for diagnostic value of long non-coding RNAs as biomarkers for ankylosing spondylitis: A systematic review and meta-Analysis.**

| **Electronic database** | **Combination** | **No of articles** | **Last searching date** |
| --- | --- | --- | --- |
| PubMed | (("long noncoding RNA" OR "long non-coding RNA" OR "long non coding RNA" OR "long ncRNA" OR "long ncRNAs" OR "ncRNAs, long" OR "lnc RNA" OR "RNA, long noncoding" OR "noncoding RNA, long" OR lncRNA OR ncRNA)) AND (("Ankylosing Spondylitis" OR "Ankylosing Spondylarthritides" OR “Ankylosing Spondyloarthritis”)) | 170 | March 15, 2025 |
| EMBASE | ('long non-coding RNA*':ti,ab,kw OR 'liRNA*':ti,ab,kw OR 'long ncRNA*':ti,ab,kw OR 'lnc RNA':ti,ab,kw OR 'ncRNAs, long':ti,ab,kw OR 'RNA, long noncoding':ti,ab,kw OR 'noncoding RNA, long':ti,ab,kw OR 'lncRNA*':ti,ab,kw) AND ('Ankylosing Spondylitis':ti,ab,kw OR 'Ankylosing Spondyloarthritis':ti,ab,kw OR 'Ankylosing Spondylarthritides':ti,ab,kw OR 'Bechterew Disease':ti,ab,kw) | 57 | March 15, 2025 |
| Scopus | (TITLE-ABS-KEY ("long noncoding RNA*" ) OR TITLE-ABS-KEY ( "long non-coding RNA*" ) OR TITLE-ABS-KEY ( "long non coding RNA" ) OR TITLE-ABS-KEY ( "long ncRNA" ) OR TITLE-ABS-KEY ( "long ncRNAs" ) OR TITLE-ABS-KEY ( "ncRNAs, long" ) OR TITLE-ABS-KEY ( "lncRNA*" ) OR TITLE-ABS-KEY ( "linRNA*" ) OR TITLE-ABS-KEY ( "ncRNA" ) OR TITLE-ABS-KEY ( "RNA, long noncoding" ) OR TITLE-ABS-KEY ( "noncoding RNA, long" ) AND TITLE-ABS-KEY ( "Ankylosing Spondylitis" ) OR TITLE-ABS-KEY ( "Ankylosing Spondyloarthritis" ) OR TITLE-ABS-KEY ( "Ankylosing Spondylarthritides" ) OR TITLE-ABS-KEY ( "Bechterew Disease")) | 58 | March 15, 2025 |
| Hinari | ((TitleCombined:("long non-coding RNA*")) OR (TitleCombined:(liRNA*)) OR (TitleCombined:(lncRNA*)) OR (TitleCombined:("long non coding RNA*"))) AND ((TitleCombined:("Ankylosing Spondylitis")) OR (TitleCombined:("Ankylosing Spondyloarthritis"))) | 17 | March 15, 2025 |
| Other sources | Articles retrieved from the reference lists of pertinent publications identified through database searches | 9 | March 15-16, 2025 |
