# Supplementary material for: Diagnostic value of long noncoding RNAs as biomarkers for Ankylosing Spondylitis: A systematic review and meta-analysis
Source: PLoS One. 2025 Jul 28;20(7):e0328249. doi: 10.1371/journal.pone.0328249 (PMC12303277; doi:10.1371/journal.pone.0328249)
Supplement: S2 Table — (DOCX) [file pone.0328249.s003.docx]

**S2 Table: A comprehensive list of all data extracted from the primary research sources for this systematic review and meta-analysis, including the names of data extractors and the dates of extraction.**

| **Authors** | **Year** | **County** | **LncRNAs** | **Expression** | **Specimen** | **Method** | **Reference** | **Participants** | | **Cut-off`** | **Sen (%)** | **Spe (%)** | **AUC** | **Name of data extractors** | **Date of data extraction** |
| --- | --- | --- | --- | --- | --- | --- | --- | --- | --- | --- | --- | --- | --- | --- | --- |
|  |  |  |  |  |  |  |  | **AS** | **HC** |  |  |  |  |  |  |
| Li X et al | 2017 | China | AK001085 | Downregulated | Serum | qRT-PCR | β-actin | 117 | 76 | 0.186 | 62.9 | 93.6 | 0.868 | EA, DA, and MAB | June 10, 2024 |
| Lan et al | 2018 | China | TUG1 | Downregulated | Serum | qRT-PCR | β-actin | 82 | 32 | NA | NA | NA | 0.796 | EA, DA, and MAB | June 10, 2024 |
| Lan et al | 2018 | China | TUG1 | Downregulated | Biopsies | qRT-PCR | β-actin | 34 | 32 | NA | NA | NA | 0.891 | EA, DA, and MAB | June 10, 2024 |
| Zhong et al | 2019 | China | LINC00311 | Upregulated | Plasma | qRT-PCR | 18S rRNA | 80 | 80 | NA | NA | NA | 0.904 | EA, DA, and MAB | June 10, 2024 |
| Liu et al | 2019 | China | MEG3 | Downregulated | Serum | qRT-PCR | β-actin | 172 | 98 | NA | NA | NA | 0.748 | EA, DA, and MAB | June 10, 2024 |
| Liu et al | 2019 | China | MEG3 | Downregulated | Biopsies | qRT-PCR | β-actin | 42 | 36 | NA | NA | NA | 0.886 | EA, DA, and MAB | June 10, 2024 |
| Zhang et al | 2020 | China | H19 | Upregulated | PBMCs | qRT-PCR | β-actin | 49 | 49 | NA | NA | NA | 0.653 | EA, DA, and MAB | June 10, 2024 |
| Zhang et al | 2020 | China | LOC101929023 | Upregulated | PBMCs | qRT-PCR | β-actin | 49 | 49 | NA | NA | NA | 0.637 | EA, DA, and MAB | June 10, 2024 |
| Wang et al | 2021 | China | 326C3.7 | Upregulated | PBMCs | qRT-PCR | β-actin | 68 | 29 | NA | 80 | 60.5 | 0.739 | EA, DA, and MAB | June 10, 2024 |
| Li M et al | 2021 | China | Lnc-ITSN1-2 | Upregulated | PBMCs | qRT-PCR | GAPDH | 63 | 60 | NA | 69.8 | 96.7 | 0.900 | EA, DA, and MAB | June 10, 2024 |
| Wang et al | 2022 | China | Linc00304 | Upregulated | PBMCs | qRT-PCR | β-actin | 24 | 20 | 0.413 | 90 | 42.3 | 0.687 | EA, DA, and MAB | June 10, 2024 |
| Wang et al | 2022 | China | Linc00926 | Upregulated | PBMCs | qRT-PCR | β-actin | 24 | 20 | 0.299 | 88.2 | 41.7 | 0.664 | EA, DA, and MAB | June 10, 2024 |
| Wang et al | 2022 | China | MIAT | Upregulated | PBMCs | qRT-PCR | β-actin | 24 | 20 | 0.432 | 89 | 44.3 | 0.623 | EA, DA, and MAB | June 10, 2024 |
| Ding et al | 2023 | China | NONHSAT227927.1 | Upregulated | PBMCs | qRT-PCR | β-actin | 50 | 30 | NA | NA | NA | 0.846 | EA, DA, and MAB | June 10, 2024 |
| Tawfeek et al | 2023 | Egypt | TUG1 | Upregulated | Plasma | qRT-PCR | U6 | 50 | 50 | 6.2 | 88 | 84 | 0.874 | EA, DA, and MAB | June 10, 2024 |
| Esawy et al | 2023 | Egypt | H19 | Upregulated | Plasma | qRT-PCR | β-actin | 53 | 53 | 1.41 | 81.1 | 100 | 0.955 | EA, DA, and MAB | June 10, 2024 |
