# Supplementary material for: Diagnostic value of long noncoding RNAs as biomarkers for Ankylosing Spondylitis: A systematic review and meta-analysis
Source: PLoS One. 2025 Jul 28;20(7):e0328249. doi: 10.1371/journal.pone.0328249 (PMC12303277; doi:10.1371/journal.pone.0328249)
Supplement: S3 Table — (DOCX) [file pone.0328249.s004.docx]

**S3 Table: List of all studies assessed for eligibility criteria.**

| S No. | Studies assessed for eligibility criteria | Included | Excluded | Reason for exclusion |
| --- | --- | --- | --- | --- |
| 1. | Zhong H, Zhong M. LINC00311 is overexpressed in ankylosing spondylitis and predict treatment outcomes and recurrence. BMC Musculoskeletal Disorders. 2019;20:1-5. | √ |  | Included |
| 2. | Lan X, Ma H, Zhang Z, Ye D, Min J, Cai F, et al. Downregulation of lncRNA TUG1 is involved in ankylosing spondylitis and is related to disease activity and course of treatment. BioScience Trends. 2018;12(4):389-94. | √ |  | Included |
| 3 | Wang J-X, Zhao X, Xu S-Q. Screening Key lncRNAs of Ankylosing Spondylitis Using Bioinformatics Analysis. Journal of Inflammation Research. 2022:6087-96. | √ |  | Included |
| 4 | Liu W, Huang L, Zhang C, Liu Z. lncRNA MEG3 is downregulated in ankylosing spondylitis and associated with disease activity, hospitalization time and disease duration. Experimental and therapeutic medicine. 2019;17(1):291-7. | √ |  | Included |
| 5 | Ding X, Liu J, Sun Y. Expression of long non‑coding RNA NONHSAT227927. 1 and its effect on the JAK2/STAT3 signaling pathway and inflammation in patients with ankylosing spondylitis. Experimental and Therapeutic Medicine. 2023;25(5):1-9. | √ |  | Included |
| 6 | Zhang X, Ji S, Cai G, Pan Z, Han R, Yuan Y, et al. H19 increases IL-17A/IL-23 releases via regulating VDR by interacting with miR675-5p/miR22-5p in ankylosing spondylitis. Molecular Therapy-Nucleic Acids. 2020;19:393-404. | √ |  | Included |
| 7 | Wang J-x, Jing F-y, Xu Y-c, Zong H-x, Chu Y-r, Wang C, et al. The potential regulatory mechanism of lncRNA 122K13. 12 and lncRNA 326C3. 7 in ankylosing spondylitis. Frontiers in Molecular Biosciences. 2021;8:745441. | √ |  | Included |
| 8 | Li M, Zhou X. Long noncoding RNA intersectin 1-2 gradually declines during adalimumab treatment, and its reduction correlates with treatment efficacy in patients with ankylosing spondylitis. Inflammopharmacology. 2021;29(5):1371-8. | √ |  | Included |
| 9 | Li X, Chai W, Zhang G, Ni M, Chen J, Dong J, et al. Down-regulation of lncRNA-AK001085 and its influences on the diagnosis of ankylosing spondylitis. Medical science monitor: international medical journal of experimental and clinical research. 2017;23:11. | √ |  | Included |
| 10 | Tawfeek G, Fetoh D. Increased LncRNA TUG1 expression level impacted ankylosing spondylitis risk, association with disability, and patients’ quality of life. Middle East Journal of Medical Genetics. 2023. | √ |  | Included |
| 11 | Esawy MM, Ebaid AM, Abd-Elhameed A, Thagfan FA, Mubaraki MA, Alazzouni AS, et al. Assessment of circulating lncRNA H19 in ankylosing spondylitis patients and its correlation with disease activity. Journal of Personalized Medicine. 2023;13(6):914. | √ |  | Included |
| 12 | Huang D, Liu J, Wan L, Fang Y, Long Y, Zhang Y, Bao B. Identification of lncRNAs associated with the pathogenesis of ankylosing spondylitis. BMC Musculoskeletal Disorders. 2021;22:1-9. |  | √ | The outcome of interest was lacking in the study |
| 13 | Sun R, Wang X, Sun X, Zhao B, Zhang X, Gong X, Wong SH, Chan MT, Wu WK. Emerging roles of long non-coding RNAs in ankylosing spondylitis. Frontiers in Immunology. 2022;13:790924. |  | √ | Review |
| 14 | EL-Zwawy AI, Soliman E, Elsayed ET, Morsy MM. Long non-coding RNA (H19) in patients with spondyloarthritis: association with disease parameters and ultrasonographic findings. Egyptian Rheumatology and Rehabilitation. 2024;51(1):19. |  | √ | The outcome of interest was lacking in the study |
| 15 | Fang Y, Liu J. Novel regulatory role of non-coding RNAs in ankylosing spondylitis. Frontiers in immunology. 2023;14:1131355. |  |  | Review |
| 16 | Sui W, Li H, He H, Xue W, Zhao X, Dai Y. Microarray analysis of long non-coding RNA expression in ankylosing spondylitis. Integrative Molecular Medicine.2015; 2(6): 378-383 |  | √ | The outcome of interest was lacking in the study |
| 17 | Zhang C, Wang C, Jia Z, Tong W, Liu D, He C, Huang X, Xu W. Differentially expressed mRNAs, lncRNAs, and miRNAs with associated co-expression and ceRNA networks in ankylosing spondylitis. Oncotarget. 2017;8(69):113543. |  | √ | The outcome of interest was lacking in the study |
| 18 | Wang H, Yang C, Li G, Wang B, Qi L, Wang Y. A review of long non-coding RNAs in ankylosing spondylitis: pathogenesis, clinical assessment, and therapeutic targets. Frontiers in Cell and Developmental Biology. 2024;12:1362476. |  | √ | Review |
| 19 | Yu HC, Huang KY, Lu MC, Huang Tseng HY, Liu SQ, Lai NS, Huang HB. Down-regulation of LOC645166 in T cells of ankylosing spondylitis patients promotes the NF-κB signaling via decreasingly blocking recruitment of the IKK complex to K63-linked polyubiquitin chains. Frontiers in Immunology. 2021;12:591706. |  | √ | The outcome of interest was lacking in the study |
| 20 | Yi L, Song C, Liu Y, Li D, Xiao T, Guo X, Wu Y. Down-regulation of long noncoding RNA HULC inhibits the inflammatory response in ankylosing spondylitis by reducing miR-556-5p-mediated YAP1 expression. Journal of Orthopaedic Surgery and Research. 2023;18(1):551. |  | √ | The outcome of interest was lacking in the study |
| 21 | Xu Z, Li H, Chen Q, Chen G. Identification of the key genes and long non‑coding RNAs in ankylosing spondylitis using RNA sequencing Corrigendum in/10.3892/ijmm. 2022.5154. International Journal of Molecular Medicine. 2019;43(3):1179-92. |  | √ | The outcome of interest was lacking in the study |
| 22 | Xie Z, Li J, Wang P, Li Y, Wu X, Wang S, Su H, Deng W, Liu Z, Cen S, Ouyang Y. Differential expression profiles of long noncoding RNA and mRNA of osteogenically differentiated mesenchymal stem cells in ankylosing spondylitis. The Journal of rheumatology. 2016;43(8):1523-31. |  | √ | The outcome of interest was lacking in the study |
| 23 | Li C, Qu W, Yang X. Comprehensive lncRNA and mRNA profiles in peripheral blood mononuclear cells derived from ankylosing spondylitis patients by RNA-sequencing analysis. Medicine. 2022;101(4):e27477. |  | √ | The outcome of interest was lacking in the study |
| 24 | Li YX, Liu T, Liang YW, Huang JJ, Huang JS, Liu XG, Cheng ZY, Lu SX, Li M, Huang L. Integrative analysis of long non-coding RNA and messenger RNA expression in toll-like receptor 4-primed mesenchymal stem cells of ankylosing spondylitis. Annals of translational medicine. 2021;9(20):1563. |  | √ | The outcome of interest was lacking in the study |
