# Supplementary material for: Diagnostic value of long noncoding RNAs as biomarkers for Ankylosing Spondylitis: A systematic review and meta-analysis
Source: PLoS One. 2025 Jul 28;20(7):e0328249. doi: 10.1371/journal.pone.0328249 (PMC12303277; doi:10.1371/journal.pone.0328249)
Supplement: Attachment — Submitted filename: COMMENTS.docx [file pone.0328249.s005.docx]

**Introduction**

- From Lines 56-65 it is better to write about how Long noncoding RNAs (lncRNAs) have emerged as promising biomarkers for various diseases, including ankylosing spondylitis (AS), a chronic inflammatory condition primarily affecting the spine and sacroiliac joints A systematic review and meta-analysis could address your interest in the diagnostic value of lncRNAs for AS. Here's a broad outline of what such a study might entail so please improve the these points
- The introduction needs revision focused what you want to write Diagnostic value of long noncoding RNAs as biomarkers for ankylosing 2 spondylitis
- The objective is better to write like To evaluate the diagnostic utility of long noncoding RNAs (lncRNAs) as biomarkers for ankylosing spondylitis (AS) by systematically reviewing and analyzing existing literature (optional not mandatory).

**2. Methods**

- **Line 111 Study Design or registration?**
- **Search Strategy**
- **Databases:** PubMed, Embase, Cochrane Library, Web of Science.
- **Keywords:** "long noncoding RNAs", "lncRNAs", "ankylosing spondylitis", "AS", "biomarkers", "diagnostic value".
- **Search period:** ?.

**Inclusion Criteria**

- **Study Type:** Case-control studies, cohort studies, and cross-sectional studies?
- **Population:** Studies involving patients diagnosed with ankylosing spondylitis.?
- **Outcome Measures:** Reports of lncRNA expression levels and their correlation with AS diagnosis.?
- **Language:** Studies published in English.?
- **Method test?**

**Exclusion Criteria**

- **Non-relevant Studies:** Studies not focused on lncRNAs or AS.
- **Low Quality:** Studies with insufficient data or methodological flaws.

**Data Extraction**

- **Study Characteristics:** Author, year, country, study design.
- **Participants:** Sample size, demographics.
- **lncRNA Details:** Specific lncRNAs studied, methods of measurement.
- **Outcomes:** Sensitivity, specificity, area under the curve (AUC), diagnostic accuracy.

**Quality Assessment**

- **Tools:** Use the Quality Assessment of Diagnostic Accuracy Studies (QUADAS-2) tool to assess risk of bias and applicability.

**Statistical Analysis**

- **Meta-Analysis:** Calculate pooled sensitivity, specificity, and diagnostic odds ratio (DOR) using random-effects models.
- **Heterogeneity:** Assess with I² statistics and chi-square tests.
- **Publication Bias:** Evaluate with funnel plots and Egger’s test.

**Results**

**Study Selection**

- **Flow Diagram:** Number of studies screened, included, and excluded.

**Study Characteristics**

- **Summary Table:** Overview of included studies, lncRNAs investigated, and their results showed that china and EGYPT. **Is only those country ?** **please see these review reference** **A review of long non-coding RNAs in ankylosing spondylitis: pathogenesis, clinical assessment, and therapeutic targets**
- **Is : Subgroup analysis of the diagnostic potential of lncRNAs in AS is possible by only two Groups ?**

**Diagnostic Performance**

- **Forest Plots:** Visual representation of sensitivity, specificity, and AUC.
- **Summary Statistics:** Pooled sensitivity, specificity, and DOR.

**Subgroup Analysis**

- **By lncRNA Type:** Different lncRNAs and their performance.
- **By Methodology:** Different diagnostic techniques or study designs.

**Heterogeneity and Bias**

- **Assessment:** Results of heterogeneity tests and bias evaluations.

**4. Discussion**

**Interpretation of Findings**

- **Clinical Relevance:** How lncRNAs perform compared to other biomarkers or diagnostic methods for AS.
- **Mechanistic Insights:** Potential roles of lncRNAs in AS pathogenesis.

**Limitations**

- **Study Quality:** Variability in study design and execution.
- **Publication Bias:** Potential impact on results.

**Future Directions**

- **Research Gaps:** Areas needing further investigation.
- **Clinical Application:** How findings could be translated into practice.
